# Comparing statistical methods in assessing the prognostic effect of biomarker variability on time-to-event clinical outcomes

**DOI:** 10.1186/s12874-022-01686-7

**Published:** 2022-07-22

**Authors:** Feng Gao, Jingqin Luo, Jingxia Liu, Fei Wan, Guoqiao Wang, Mae Gordon, Chengjie Xiong

**Affiliations:** 1 Division of Public Health Sciences, Department of Surgery, Washington University School of Medicine in St Louis, 660 S. Euclid Ave., St. Louis, MO 63110 USA; 2 Division of Biostatistics, Washington University School of Medicine in St Louis, 660 S. Euclid Ave., St. Louis, MO 63110 USA; 3 Department of Ophthalmology & Visual Sciences, Washington University School of Medicine in St Louis, 660 S. Euclid Ave., St. Louis, MO 63110 USA

**Keywords:** Patient-specific variance, Survival data, Longitudinal data, Joint model, Landmark analysis

## Abstract

**Background:**

In recent years there is increasing interest in modeling the effect of early longitudinal biomarker data on future time-to-event or other outcomes. Sometimes investigators are also interested in knowing whether the variability of biomarkers is independently predictive of clinical outcomes. This question in most applications is addressed via a two-stage approach where summary statistics such as variance are calculated in the first stage and then used in models as covariates to predict clinical outcome in the second stage. The objective of this study is to compare the relative performance of various methods in estimating the effect of biomarker variability.

**Methods:**

A joint model and 4 different two-stage approaches (naïve, landmark analysis, time-dependent Cox model, and regression calibration) were illustrated using data from a large multi-center randomized phase III trial, the Ocular Hypertension Treatment Study (OHTS), regarding the association between the variability of intraocular pressure (IOP) and the development of primary open-angle glaucoma (POAG). The model performance was also evaluated in terms of bias using simulated data from the joint model of longitudinal IOP and time to POAG. The parameters for simulation were chosen after OHTS data, and the association between longitudinal and survival data was introduced via underlying, unobserved, and error-free parameters including subject-specific variance.

**Results:**

In the OHTS data, joint modeling and two-stage methods reached consistent conclusion that IOP variability showed no significant association with the risk of POAG. In the simulated data with no association between IOP variability and time-to-POAG, all the two-stage methods (except the naïve approach) provided a reliable estimation. When a moderate effect of IOP variability on POAG was imposed, all the two-stage methods underestimated the true association as compared with the joint modeling while the model-based two-stage method (regression calibration) resulted in the least bias.

**Conclusion:**

Regression calibration and joint modelling are the preferred methods in assessing the effect of biomarker variability. Two-stage methods with sample-based measures should be used with caution unless there exists a relatively long series of longitudinal measurements and/or strong effect size (NCT00000125).

**Supplementary Information:**

The online version contains supplementary material available at 10.1186/s12874-022-01686-7.

## Background

As the field of precision medicine continues to advance, methodologies that utilize individual patient information over time are becoming increasingly valuable to provide more accurate prognosis as early as possible. In many clinical trials and epidemiologic studies, information is gathered on both repeated measures of biomarkers (longitudinal data) and clinical outcomes. There is increasing interest in modeling the longitudinal data and assessing its association with future outcomes. Various methods have been proposed for this goal. The most popular ones in medical reports are two-stage methods where sample-based descriptive statistics such as mean are first calculated for each individual and subsequently used as covariates (i.e., assumed known and free of measurement error) into conventional models such as linear regression or Cox proportional hazard model for a clinical outcome. Several studies have compared different statistical methods that relate the characteristics derived from early longitudinal data to future clinical outcomes. For example, Tu et al. [1] examined various approaches (tracing the z-scores, life-course plots and models, life-course path analysis, conditional body size analysis, multilevel analysis, latent growth curve models and growth mixture models) for the repeated measurements of weights from early ages to assess its relationship with blood pressure at age 19. Sayers et al. [2] using simulated data compared 4 different two-stage methods with a joint model in relating childhood growth to adult blood pressure. They concluded that sample-based summary measures tend to result in biased estimation for the association, while multilevel growth model (model-based two-stage or regression calibration) and joint modeling lead to unbiased result. Sweeting et al. [3] assessed the predictive ability of repeated blood pressure for the time-to-cardiovascular disease outcome. They compared various naive methods (baseline-only model, last observation carry-over forward, and cumulative mean) against more complex models (regression calibration (RC), risk-set RC, and joint modeling), but only observed a modest improvement in terms of discrimination and calibration as compared to the baseline-only model. Crozier et al. [4] also discussed recent development in methods to characterize the prenatal and early growth and its impact on whole body bone mass at age 6, though they focused more on complex model-based approaches.

All the above studies focus on the mean level or trajectory of the longitudinal measurements. In some studies, however, it is also of interest to know whether the variability (or stability) of a biomarker is predictive of outcomes. In an ophthalmology study based on subjects with at least 3-year follow-up, for example, Caprioli and Colman [5] found that long-term IOP fluctuation (measured as standard deviation of each individual’s longitudinal IOP) is associated with visual field progression in patients with POAG. Segar et al. [6] revealed that the variability in kidney function and serum electrolyte indices is independently associated with worse clinical outcomes in patients with chronic stable heart failure. Muntner et al. [7] and Whittle et al. [8] assessed data from the ALLHAT Pragmatic Trial and found that the visit-to-visit variability of blood pressure is an independent predictor for cardiovascular disease and chronic renal disease outcomes, respectively. Recently, it was also reported that cognitive variability predicts the onset of Alzheimer disease dementia [9]. Despite the recent development of joint models [10, 11], two-stage approach remains the mainstream in medical applications for assessing the effect of within-subject biomarker variability on clinical outcomes. To the best of our knowledge, however, no papers have yet compared the relative performance of various methods under this setting.

In this paper, several two-stage methods and a joint model were illustrated using data from a large multi-center randomized phase III trial, the Ocular Hypertension Treatment Study (OHTS), regarding the association between the variability of intraocular pressure (IOP) and the development of primary open-angle glaucoma (POAG). We also compared the relative performance of two-stage methods using simulated data where the association between longitudinal and survival data was introduced via underlying, unobserved, and error-free parameters including subject-specific variance [10, 11]. The model parameters for simulation were selected based on the analysis of OHTS data. This paper is organized as follows. Section 2 describes the OHTS data, model constructions, and computational implementation. Section 3 presents the results to illustrate various models using OHTS data and compares their performance by simulations. Finally, Sect. 4 concludes with a discussion.

## Methods

### Motivating example: Ocular Hypertension Treatment Study (OHTS)

OHTS is a large multi-center randomized phase III trial to evaluate the safety and efficacy of topical ocular hypotensive medication in delaying the onset of primary open-angle glaucoma (POAG) which is among the leading causes of blindness in US and worldwide. 1636 subjects were randomized to either observation or treatment with ocular hypotensive medication and followed for a median of 78 months. A prediction model to identify ocular hypertensive subjects who are at high risk for developing POAG has been developed from OHTS data and been widely used in the ophthalmology community [12]. The prediction model includes 5 baseline factors—age, intraocular pressure (IOP), central corneal thickness (CCT), pattern standard deviation (PSD), and vertical cup to disc ratio (VCDR). Among these factors, IOP is the only factor that can be modified by current treatment. As of today, many studies have confirmed that a decrease in mean IOP level can reduce the risk of developing POAG. There is still controversy about the impact of IOP variability on POAG.

**Fig. 1:**
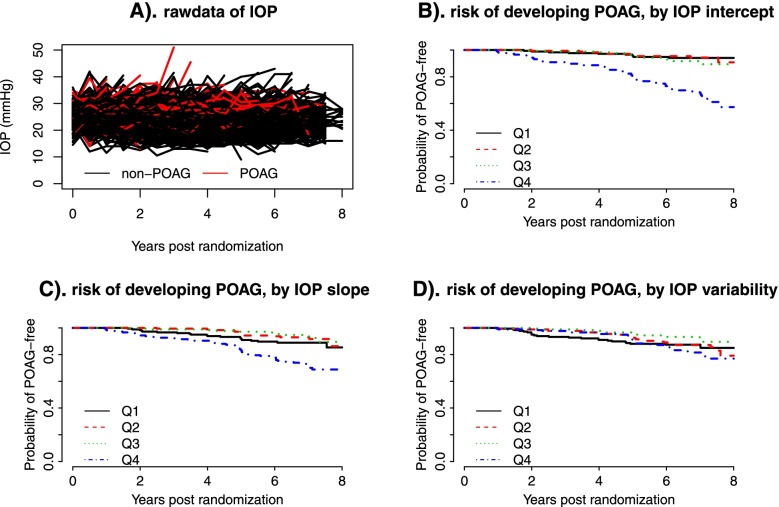
An exploratory analysis to assess the association between IOP-derived subject-specific characteristics (i.e., intercept, slope, and variance of residuals from the OLS model of IOPs against measurement times) and the risk of developing POAG using the OHTS data. **A** raw data of longitudinal IOP over follow-up time; **B** risk of developing POAG by the quartiles of IOP intercept; **C** risk of developing POAG by the quartiles of IOP slope; **D** risk of POAG by the quartiles of within-subject IOP variability

The analysis cohort for the current study consisted of 709 participants who were randomized to the observation arm, had completed at least two post-randomization visits (i.e., with at least 3 IOP measurements), and had complete baseline data for age, IOP, CCT, PSD, and VCDR. The eye-specific predictors (IOP, CCT, PSD, VCDR) were based on the first eye with POAG or a randomly selected eye in participants without POAG. The primary endpoint was time from randomization to the POAG onset. Those subjects who did not develop POAG were censored at the date of study closeout. These baseline predictors were standardized to have mean 0 and variance 1 and the coefficients from the regression models represented the effect per 1-SD change. The objective of current study is to compare the performance of different statistical methods, and a detailed interpretation on the effect of IOP variability for the risk of POAG has been described in Gordon et al. [13]. An exploratory analysis was performed by fitting a simple linear regression model of IOPs against measurement times to each subject to estimate intercept, slope and (logarithm transformed) variance of residuals. Figure 1 explored the relationship between these IOP-derived characteristics (which were further categorized into quartiles) and the risk of developing POAG. There was a dramatically increased risk of developing POAG in the 4^th^ quartile of IOP intercept (Fig. 1B). Similar trend was shown in IOP slope though the relationship may not be linear (Fig. 1C), while a substantial overlap was noted across quartiles of the within-subject IOP variability (Fig. 1D).

### Models for data analysis and simulation

Data were analyzed by a joint model of longitudinal and survival data, as well as 4 different two-stage models to assess the association between longitudinal and survival outcomes, especially for the effect of biomarker variability.

### Notations

Suppose there were N subjects. Let T_i_ = min(D_i_, C_i_) be the observed survival time for the i^th^ subject, where D_i_ was the potential failure time and C_i_ was the potential censoring time independent of D_i_. Let Δ_i_ be the corresponding censoring indicator, with Δ_i_ = 1 if T_i_ = D_i_ and Δ_i_ = 0 otherwise. Let Y_ij_ denote the longitudinal data for i^th^ individual at time t_ij_, and the longitudinal data only included those measurements taken prior to the time of event or censoring. Finally, we denoted the baseline covariates predictive of longitudinal and survival processes as X_i_ and Z_i_ respectively, which may or may not be the same.

#### Joint model assessing biomarker variability on survival outcome

The joint model consists of two sub-models, a measurement model for longitudinal data and an intensity model for survival data, and the two sub-models are associated via shared latent random effects including subject-specific variance [14]. Specifically,

- the measurement sub-model describes the trajectory of longitudinal data using a linear mixed model,
  1 $${Y}_{ij}={\beta }_{0}+{\beta }_{1}{t}_{ij}+{\beta }_{2}{X}_{i}+ {I}_{i}+{S}_{i}{t}_{ij}+{e}_{ij}$$
  $$e_{ij}\sim N\left(0,\sigma_{vi}^2\right)and\;\log\left(\sigma_{vi}^2\right)=\mu_V+U_i$$
  $$\begin{pmatrix}I_i\\S_i\\U_i\end{pmatrix}\sim N\left(\begin{pmatrix}0\\0\\0\end{pmatrix},\Sigma\right),and\;\Sigma=\begin{pmatrix}\sigma_I^2&\rho_{12}\sigma_I\sigma_S&\rho_{13}\sigma_I\sigma_V\\\rho_{12}\sigma_I\sigma_S&\sigma_S^2&\rho_{23}\sigma_S\sigma_V\\\rho_{13}\sigma_I\sigma_V&\rho_{23}\sigma_S\sigma_V&\sigma_V^2\end{pmatrix}$$
- where β_0_ and β_1_ represents intercept and slope, respectively, and β_2_ is a vector of parameters for other baseline covariates. I_i_ and S_i_ are the subject-specific random intercept and slope as in a conventional linear mixed model [15]. ${e}_{ij}$ is the error for subject i at time j, independently following a normal distribution with a mean zero and variance ${\sigma }_{vi}^{2}$. We assume a log-linear relationship between the overall variance, ${\mu }_{V}$, and the subject-specific random variance, ${U}_{i}$. {${I}_{i}{, S}_{i}, {U}_{i}$} is the vector of random effects with its distribution defined as i.i.d. multivariate normal with a mean vector {0, 0, 0} and a symmetric and positive-definite variance–covariance ∑. {${\sigma }_{I}^{2},{\sigma }_{S}^{2},{\sigma }_{V}^{2}$} is the vector of variance parameters of ${I}_{i}{, S}_{i}, and {U}_{i}$. {${\rho }_{12}, {\rho }_{13}, {\rho }_{23}$} is the vector of correlation parameters among the random effects. That is, unlike the conventional linear mixed models, model (1) explicitly allowed subject-specific variance. The above model is similar to our previous joint model [10] except that its variance–covariance matrix has been extended following Leckie et al. [16] to allow exploring the full correlation among all subject-specific random effects.
- the intensity sub-model is a semi-parametric piecewise exponential distribution,
  2 $${\lambda }_{i}\left(t\right)={\lambda }_{0}\left(t\right)\mathrm{exp}\left({\alpha }_{0}+{\alpha }_{1}{Z}_{i}+{\gamma }_{1}{I}_{i}+{\gamma }_{2}{S}_{i}+{\gamma }_{3}{U}_{i}\right)$$
- where λ_0_(t) is the baseline hazard when all covariates are 0. The subject-specific random effects $I_i,S_i,and\;U_i$ are from model (1) and thus induced association between the longitudinal and the survival process, with ${\gamma }_{3}$ specifically for the effect of wthin-subject biomarker variability. α_0_ denotes intercept and α_1_ is a vector of parameters for other baseline covariates. To obtain a flexible estimate to λ_0_(t), we split the survival time into K subintervals (with a known value for K) and assume λ_0_(t) being a step-function with height $\lambda_{k}$ at each interval $(t_{k} ,t_{k + 1} )$, for k = 1, 2, …, K.

The full joint distribution is specified in the form of,

$$\mathrm{f}(\mathrm{Y},\mathrm{ T},\uptheta ) =\mathrm{ f}(\mathrm{Y}|\uptheta )\mathrm{ f}(\mathrm{T}|\mathrm{Y},\uptheta )\mathrm{ f}(\uptheta )$$

with θ = {β, α, γ, $\mu_{V}$,$\lambda_{k}$, $\sigma_{I}^{2}$, $\sigma_{S}^{2}$, $\sigma_{V}^{2}$, ${\rho }_{12}$, ${\rho }_{13}$,${\rho }_{23}$} the unknown parameters as given in models (1) and (2), and estimated under a Bayesian framework using Markov Chain Monte Carlo (MCMC) method [17]. The following prior distributions are specified for the unknown parameters,

$$\mathrm\alpha,\mathrm\beta,\mathrm\gamma,{\mathrm\mu}_{\mathrm v}\sim\mathrm N(0,100),$$

$$\lambda_{k} \sim {\text{G}}\left( {0.1,\;1} \right),\;{\text{and}}\;\sum { \sim {\text{IW}}\left( {\left( {\begin{array}{*{20}c} {0.01} & 0 & 0 \\ 0 & {0.01} & 0 \\ 0 & 0 & {0.01} \\ \end{array} } \right)} \right)} \,$$

where $N(a,b)$ denoted a Normal distribution with mean *a* and variance *b*, $G(a,b)$ represented a Gamma distribution with mean a/b and variance a/b^2^, and IW represented an inverse-Wishart distribution which is an inverse of 3-dimension Gamma distribution. The resultant posterior distributions are a multivariate normal distribution for the measurement sub-model and a Poisson distribution for the intensity sub-model at the k^th^ interval $(t_{k} ,t_{k + 1} )$.

#### Two-stage models assessing biomarker variability on survival outcome

In a two-stage analysis, only longitudinal data are analyzed in the first stage to estimate summary statistics for each individual, and these summary statistics are then used as covariates in the regression models in the second stage. In this study, 4 different two-stage models are considered.

1. Simple approach (Naïve): In the first stage, a simple ordinary least square (OLS) regression model is fitted to each subject separately and includes measurements from all available visits to estimate intercept ($${I}_{i}$$), slope ($${S}_{i})$$, and the variance of residuals ($${U}_{i})$$ for i^th^ subject. In the second stage, the above estimates are used as covariates in a Cox proportional hazards model to assess their prognostic effects on the time to POAG, where the hazard function for i^th^ subject is defined as,
  $$\lambda_i\left(t\right)=\lambda_0\left(t\right)\exp\left(\alpha_1Z_i+\gamma_1I_i+\gamma_2S_i+\gamma_3U_i\right).$$
2. Landmark analysis (LMA): A set of visits are first selected as landmark points. In the first stage, a series of OLS models are fitted to each individual. Each OLS model includes longitudinal measurements accumulated up to the given landmark point to estimate intercept ($${I}_{il}$$), slope ($${S}_{il})$$, and the variance of residuals ($${U}_{il})$$ for i^th^ subject at l^th^ landmark point. In the second stage, these estimates are used as fixed covariates in a series of conventional Cox models and each model is fitted at a given landmark point onwards (i.e., including participants still at risk of developing an endpoint). The hazard function for i^th^ subject at l^th^ landmark point is defined as,
  $$\lambda_{il}(t)=\lambda_{0l}(t)exp(\alpha_1Z_i+\gamma_1I_{il}+\gamma_2S_{il}+\gamma_3U_{il})$$
  where ${\lambda }_{0l}$ represents the unspecified baseline hazard function at l^th^ landmark point and {${\gamma }_{1}$,${\gamma }_{2}$,${\gamma }_{3}$}is the vector of average effects across landmark points. {${\gamma }_{1}$,${\gamma }_{2}$,${\gamma }_{3}$} is estimated similar to that of Van Houwelingen [18] by stacking the datasets across all landmark points and using robust sandwich variance for the precision of estimation. One main difference is that we fitted a stratified (stratified by landmark points) Cox model and left the baseline hazard ${\lambda }_{0l}\left(t\right)$ totally unspecified, while Van Houwelingen [18] adapted a delayed entry to ensure a uniform time scale across all models and thus to estimate baseline hazard function.
3. Cox model with time-dependent covariates (td-Cox): In the first stage, a series of OLS models are fitted in a similar way as the above LMA to estimate intercept ($${I}_{il}$$), slope ($${S}_{il})$$, and the variance of residuals ($${U}_{il})$$ for i^th^ subject at l^th^ visit. In the second stage, their effects on POAG are assessed using a Cox proportional hazards model with time-dependent covariates, and the hazard function for i^th^ subject is defined as,
  $$\lambda_i(t)=\lambda_0(t)exp(\alpha_1Z_i+\gamma_1I_i(t)+\gamma_2S_i(t)+\gamma_3U_i(t))$$
  where $\left\{{I}_{i}\left(t\right),{S}_{i}\left(t\right), {U}_{i}\left(t\right)\right\}$ represents time-varying covariates incorporated via a counting process style [19] for {${I}_{il}$, ${S}_{il}, {U}_{il}$} at different visits.
4. Regression Calibration (RC): this approach has been widely used in the measurement error framework where model-based (rather than sample-based) estimates are used as covariates in the regression analysis in the second stage to assess the association between biomarker-derived characteristics and clinical outcome [2, 3]. Specifically, RC is essentially a two-stage implementation of the joint model as specified in Sect. 2.2.1. In the first stage, Model (1) is fitted to all subjects to estimate the subject-specific intercept ($${I}_{i}$$), slope ($${S}_{i})$$, and the variance of residuals ($${U}_{i}).$$ In the second stage, these model-based estimates are used as covariates into a Cox model, where the hazard function for i^th^ subject is defined as,
  $$\lambda_i(t)=\lambda_0(t)exp(\alpha_1Z_i+\gamma_1I_i+\gamma_2S_i+\gamma_3U_i)$$
  For comparison with the joint model (the true model used for data simulating later on), logarithm transformed variance of residuals from OLS was actually used as the measure of within-subject variability throughout this paper. Note that at least 3 repeated measurements are needed in order to fit an OLS model in a given subject. In the analysis of OHTS data, only those subjects who had at least 3 measurements were included in fitting these models, even though this restriction was not required for the joint model and RC for a fair comparison. In the simulation studies, for each subject we set a minimum lead-in time (i.e., 1 year) where we started to collect longitudinal measurements prior to the time-0 of survival model and thus avoided excluding any simulated subjects from analysis.

### Computational implementation

The unknown parameters θ = {β, α, γ, ${\mu }_{V}$, ${\lambda }_{k}$, $\sigma_{I}^{2}$, $\sigma_{S}^{2}$, $\sigma_{V}^{2}$, ${\rho }_{12}$, ${\rho }_{13}$,${\rho }_{23}$} in the joint model and RC were fitted using the Gibbs sampling built into the software WinBUGS (http://www.mrc-bsu.cam.ac.uk/bugs/). The parameter estimates and standard errors were calculated as the mean and standard deviation of the posterior samples from Gibb’s sampling. The estimation procedure was implemented using the R2WinBUGS library in the statistical package R [20]. We used three parallel MCMC sampling chains with different starting values. The convergence of each chain was monitored by the trace plots and the diagnostic statistics of Gelman et al. [17]. Following recommendations by Goldstein et al. [21] for hierarchical models with random effect in level-1 variance, we took a relatively long adaptation period for MCMC. In the analysis of OHTS data, the posterior mean of the parameters were based on 25,000 iterations following a 25,000-iteration of burn-in period. In the simulated data, we used 15,000 iterations following a 15,000-iteration of burn-in period to reduce computational time. All the other two-stage models were fitted using the survival library in the statistical package R.

## Results

### Ocular Hypertension Treatment Study (OHTS)

The OHTS analysis cohort included 709 participants with a median of 14 IOP measurements (range 3–17) and a median follow–up of 6.9 years (range 1.0–8.1). A total of 97 participants developed POAG endpoints during follow-up. The OHTS data were analyzed by joint model and two-stage methods, adjusting baseline factors (age, CCT, and VCDR) in both longitudinal (IOP) and survival (POAG) sub-models (R codes for RC and joint model listed in the Supplementary Materials). In LMA, a total of 9 visits, semi-annually at time = {1, 1.5, 2, 2.5, 3, 3.5, 4, 4.5, 5}, were selected as the landmark points. Table 1 showed the estimated parameters ($\widehat{\theta }$) and standard error (SE) from the joint model and the two-stage methods. Means and standard deviations for the sample-based estimates {I_i_, S_i_, U_i_} from the Naïve, LMA, and tdCox models were also included in Table 1. Because each subject had series of estimates across different landmark points (visits) in LMA and tdCox, the estimates at 3-year (i.e., the median landmark point) were presented for illustration. As expected, the estimates from the sample-based two-stage models showed relatively large SDs especially in the IOP slope and within-subject IOP variability.

- In the longitudinal sub-model of joint model, we observed significant between-subject variation in the random intercepts, slopes, as well as the within-subject fluctuation. There was a weak but significant decrease of IOP over time (with slope ${\beta }_{1}=-0.17$ mmHg/year), and the change varied considerably between participants, i.e., with a standard deviation of random slope ${\sigma }_{S}=0.41$ as comparing to its average trajectory (${\beta }_{1}=-0.17).$ The random intercept and within-subject variation also varied significantly across participants, but the magnitude of standard deviations (${\sigma }_{\mathrm{I}}=2.51$ for random intercept and ${\sigma }_{V}=0.67$ for within-subject variability) were not so big as comparing to the corresponding mean levels (${\beta }_{0}=24.55 and {\mu }_{V}=1.64).$ The analysis revealed only weak correlation between intercept and slope $({\rho }_{12}=0.13)$, intercept and variation $({\rho }_{13}=0.18)$, as well as between slope and variation (${\rho }_{23}=0.24$).
- The survival sub-model of joint model showed baseline hazards function of λ_0_(t) = {0.001 ± 0.001, 0.003 ± 0.002, 0.006 ± 0.002, 0.014 ± 0.004, 0.017 ± 0.005, 0.011 ± 0.004, 0.043 ± 0.015} at time intervals t = {0, 2, 3, 4, 5, 6, 7, ∞}, respectively. The model indicated that those subjects with high mean IOP or large slope had a significantly increased risk to develop POAG (γ_1_ = 0.23, 95% credit interval (CI): 0.12, 0.34; γ_2_ = 1.19, 95% CI: 0.23, 2.24), and there was no significant association between IOP variability and POAG (${\gamma }_{3}$=0.12, 95% CI: -0.32, 0.57).
- Among these two-stage methods, RC reached exactly the same conclusion as the joint model, while all other 3 methods showed that only IOP intercept was significantly associated with POAG. One reason for the inconsistent results is that sample-based two-stage models are more sensitive to the number of data points in the longitudinal IOPs when calculating subject-specific estimates, especially for slope (S_i_) and variability (U_i_). For a subject with very short IOP series, for example, one may end up with low S_i_ (and/or U_i_) because a short IOP series does not allow enough room for fluctuation. On the other hand, one may end up with high S_i_ (and/or U_i_) because a short IOP series is more sensitive to potential outliers. Consequently, this could result in non-linear relationship (as shown in Fig. 1) and decreased statistical power. In contrast, a model-based two-stage methods such as RC allows borrowing information from similar subjects and leads to more reliable subject-specific estimates. An exploratory analysis similar to that of Fig. 1 was performed using subject-specific estimates from RC and the assumption of linearity was well satisfied in all estimates (Supplementary Fig. 1). An exploratory analysis was also performed regarding the precision of estimation and it revealed that a relatively long series of IOPs is needed in order to reliably estimate the within-subject variability (Supplementary Materials).
- Two sensitivity analyses were further performed using the OHTS data. The first sensitivity analysis compared a joint model with an exponential survival sub-model to the one with a piecewise exponential survival. The estimated parameters were listed side by side for an easy comparison and very small differences were found (Supplementary Table 1). In the second sensitivity analysis, the two-stage models were defined using both Bayesian and non-Bayesian paradigm, and the estimated parameters were very close under both framework (Supplementary Table 2). Therefore, to reduce computational time in the subsequent simulation study, the data were generated from a joint model with exponential survival and all the sample-based two-stage models were analyzed using standard software under non-Bayesian framework.

**Table 1:** Estimated parameters ($\widehat{\theta }$) and its standard error (SE) for the association between the longitudinal intraocular pressure (IOP) and the risk of developing primary open-angle glaucoma (POAG) based on the OHTS data

Parameters	Joint Model		RC		Naive		LMA^a^		tdCox^a^	
	$\widehat{\theta }$	SE	$\widehat{\theta }$	SE	$\widehat{\theta }$	SE	$\widehat{\theta }$	SE	$\widehat{\theta }$	SE
**Longitudinal model:**										
**Fixed effects**										
Intercept (${\beta }_{0})$	24.55*	0.108	24.57*	0.109	24.60		24.65		24.65	
Slope (${\beta }_{1})$	-0.169*	0.021	-0.179*	0.021	-0.037		-0.180		-0.180	
Age (decades)${(\beta }_{2})$	0.244*	0.106	0.235*	0.112	–		–		–	
CCT ${(\beta }_{3})$	0.045	0.108	0.031	0.103	–		–		–	
VCD ${(\beta }_{4})$	-0.063	0.110	-0.071	0.110	–		–		–	
**Random effects**										
SD of Intercept (${\sigma }_{I})$	2.514*	0.088	2.518*	0.088	3.098		3.031		3.031	
SD of slope (${\sigma }_{S})$	0.412*	0.021	0.410*	0.021	1.393		1.664		1.664	
SD of variation (${\sigma }_{V})$	0.668*	0.027	0.668*	0.027	0.905		1.012		1.012	
Mean of variation (${\mu }_{V})$	1.641*	0.031	1.639*	0.030	2.185		1.090		1.090	
Correlation (${\rho }_{12})$	0.131*	0.060	0.113*	0.059	–		–		–	
Correlation (${\rho }_{13})$	0.183*	0.051	0.181*	0.050	–		–		–	
Correlation (${\rho }_{23})$	0.235*	0.059	0.228*	0.060	–		–		–	
**Survival model:**										
**Baseline factor**										
Age (decades)${(\alpha }_{1})$	0.216	0.125	0.182	0.110	0.082	0.107	0.114	0.109	0.102	0.107
CCT ${(\alpha }_{2})$	-0.639*	0.129	-0.644*	0.113	-0.672*	0.110	-0.643*	0.129	-0.662*	0.112
VCD ${(\alpha }_{3})$	0.543*	0.142	0.526*	0.116	0.549*	0.111	0.425*	0.112	0.517*	0.112
**Effects of follow-up IOP**										
Intercept (${\gamma }_{1})$	0.226*	0.056	0.275*	0.047	0.212*	0.034	0.161*	0.038	0.203*	0.034
Slope (${\gamma }_{2})$	1.190*	0.514	0.852*	0.348	0.038	0.054	0.017	0.048	0.026	0.051
Variation (${\gamma }_{3})$	0.121	0.228	0.103	0.183	-0.052	0.094	0.108	0.084	0.071	0.084

^*^P < 0.05; *RC* Regression calibration; Naïve: simple OLS; *LMA* Landmark analysis, *td-Cox*: time-dependent Cox model

^a^Based on the subject-specific intercept, slope, and logarithm transformed variance of residuals estimated at the 3-year landmark point

### Simulation study

Two sets of simulations were conducted to assess the relative performance of the two-stage methods. The first simulation was designed to assess the impact of the number of longitudinal measurements on detecting the association between within-subject variability and survival outcome. The second simulation assessed the influence of random effects on estimation. Data were generated from the joint model with an exponential survival distribution, and censoring times were introduced from a uniform distribution U [1, 8] that was independent of survival time. Model parameters θ = {β, α, γ, ${\mu }_{V}$, ${\sigma }_{I}^{2}$, $\sigma_{S}^{2}$, ρ_12_, ρ_13_, ρ_23_} were modified after the OHTS joint model. The model performance was summarized as the bias of the estimated parameters relative to the true values. Each simulated scenario included 100 samples (mainly due to intensive computation in the joint model and the regression calibration model) and each sample included 500 subjects with approximately 80% censoring rate.

#### Impact of frequency and number of longitudinal measurements

The data were simulated under a total of 16 scenarios based on the combinations of 4 conditions (see Table 2): the frequency of measurements (semi-annually vs. quarterly), the minimum lead-in times (1-year vs. 3-year), the average IOP change over time (β_1_ = 0 vs. β_1_ = -0.5), and the strength of association between within-subject IOP variability and POAG (γ_3_ = 0 vs. γ_3_ = 0.5). Specifically,

- The number of longitudinal measurements were determined by the frequency of visits and lead-in time. For a trial with semi-annual frequency, for example, the longitudinal data were generated at times t = {-1, -0.5, 0, 0.5, 1.0, 1.5, 2.0, 2.5, 3.0, 3.5, 4.0, 4.5, 5.0} given 1-year lead-in time and at times t = {-3, -2.5, -2, -1.5, -1, -0.5, 0, 0.5, 1.0, 1.5, 2.0, 2.5, 3.0, 3.5, 4.0, 4.5, 5.0} given 3-year lead-in time. For simplicity, in the simulation we assume all subjects with equal-spaced measurements, but this is not necessary in general as a linear regression has been used to estimate subject-specific summary statistics.
- A baseline covariate (X) was generated from a standard normal distribution N(0,1), and the covariate was assumed to have effect on both survival time (α_1_ = 0.5) and longitudinal data (β_2_ = 1.0). All other parameters remained constant across all simulated scenarios, with β_0_ = 25, γ_1_ = 0.2, γ_2_ = 1.0, ${\sigma }_{I}$=2.5, ${\sigma }_{S}$=0.4,${\mu }_{V}$ =1.5, ${\sigma }_{V}$=0.7, ${\rho }_{12}$=0.1, ${\rho }_{13}$=0.2, and ${\rho }_{23}$=0.2.

**Table 2:** Average of estimated effect of biomarker variability ($\widehat{{\gamma }_{3}}$) on survival outcome and its standard error (SE) from the joint model and two-stage methods based on the first simulation

Simulated scenarios					naive		LMA		td-Cox		RC		Joint model	
Frequency of visits	Min #visits (lead-in time)	β_1_	${\gamma }_{3}$	Scenario#	$\widehat{{\gamma }_{3}}$	SE	$\widehat{{\gamma }_{3}}$	SE	$\widehat{{\gamma }_{3}}$	SE	$\widehat{{\gamma }_{3}}$	SE	$\widehat{{\gamma }_{3}}$	SE
Semi-annually	3 (1-yr)	0.0	0.0	1	-0.353	0.053	0.002	0.034	0.001	0.042	0.039	0.129	0.011	0.150
			0.5	2	-0.238	0.060	0.067	0.038	0.076	0.046	0.372	0.128	0.507	0.154
		-0.5	0.0	3	-0.355	0.054	0.002	0.035	0.003	0.042	0.018	0.127	-0.017	0.149
			0.5	4	-0.226	0.060	0.074	0.039	0.088	0.045	0.419	0.126	0.528	0.161
	7 (3-yr)	0.0	0.0	5	-0.129	0.081	-0.019	0.081	-0.018	0.075	0.044	0.117	0.002	0.129
			0.5	6	0.197	0.084	0.243	0.085	0.259	0.078	0.483	0.115	0.499	0.132
		-0.5	0.0	7	-0.121	0.081	-0.014	0.080	-0.005	0.075	0.003	0.117	-0.010	0.127
			0.5	8	0.191	0.084	0.243	0.083	0.258	0.077	0.452	0.121	0.521	0.136
Quarterly	5 (1-yr)	0.0	0.0	9	-0.214	0.083	-0.006	0.068	-0.012	0.069	0.002	0.114	-0.015	0.124
			0.5	10	0.122	0.086	0.218	0.073	0.232	0.072	0.452	0.115	0.489	0.129
		-0.5	0.0	11	-0.222	0.084	0.004	0.068	-0.013	0.069	0.017	0.112	0.011	0.124
			0.5	12	0.102	0.086	0.205	0.073	0.222	0.072	0.413	0.113	0.524	0.131
	13 (3-yr)	0.0	0.0	13	-0.080	0.090	-0.014	0.097	-0.025	0.087	0.008	0.107	0.005	0.113
			0.5	14	0.299	0.092	0.313	0.098	0.321	0.088	0.474	0.109	0.515	0.115
		-0.5	0.0	15	-0.074	0.091	-0.024	0.096	-0.023	0.087	0.001	0.108	-0.005	0.111
			0.5	16	0.302	0.091	0.314	0.098	0.321	0.088	0.462	0.108	0.519	0.119

*RC* Regression calibration; Naïve: simple OLS; *LMA* Landmark analysis, *td-Cox* Time-dependent Cox model

The simulated data were analyzed by the joint model and two-stage methods. The visits at time = {0, 1, 2, 3, 4, 5} were selected as landmark points in LMA and also used as the time intervals to calculate time-dependent covariates in td-Cox. The average estimated effect of biomarker variability ($\widehat{{\gamma }_{3}}$) and its standard error (SE) were presented in Table 2 and summarized below. All other estimated parameters were presented in the Supplementary Table 4.

- Naïve showed the worst performance among all comparison models, especially under scenarios with short lead-in time or low measurement frequency. Given 1-year lead-in time, for example, it showed a significant negative association ($\widehat{{\gamma }_{3}}<0$) when there was actually no association (γ_3_ = 0), and it led to severe underestimation when there existed a moderate association (γ_3_ = 0.5).
- LMA had a better performance as comparing to Naïve. It resulted in a reliable estimation when there was no association (γ_3_ = 0). When there existed an association (γ_3_ = 0.5), however, LMA also suffered from severe underestimation under scenarios with short lead-in time and/or low measurement frequency.
- td-Cox had almost identical performance as LMA under all simulated scenarios. This was consistent to what was reported in Putter and Van Houwelingen [22]. They studied the relationship between regression coefficients obtained in time-dependent Cox model and landmark analysis, and found that the two models are well agreed when there is no rapid change over time in the time-varying effects.
- RC had the best performance among all two-stage methods. This observation was consistent to the findings in Sayers et al. [2] that model-based two-stage methods have a much better performance than sample-based ones. The results also showed that a relatively long series of longitudinal biomarkers are needed when the effect of within-subject biomarker variability is of interest. For example, at least 7 measurements were needed in order to produce an estimation with less than 10% bias using RC.

#### Impact of the random effects from longitudinal data

Data in the second simulation were generated using the same models as the first simulation, except that the interest is to explore model performance under different magnitude of underlying variance–covariance components. Specifically,

- Data were simulated under the following 4 scenarios with a non-factorial design, assuming all the correlation coefficients ${\rho }_{12}$= ${\rho }_{13}$= ${\rho }_{23}$=0. Values in the parentheses were selected based on OHTS model and held constant when data were simulated under other scenarios.
  1. $\sigma_I$(SD of random intercept): 0.5, 1.5, (2.5), 3.5, 5.0;
  2. $\sigma_S$(SD of random slope): 0.1, 0.2, (0.4), 0.7, 1.0;
  3. $\sigma_V$(SD of within-subject variability): 0.4, (0.7), 1.0, 1.5, 2.5;
  4. $\mu_V$(Mean of within-subject variability): 0.5, 1.0, (1.5), 2.0, 3.0.
- In all the simulated scenarios, we assumed a moderate assocation between IOP variability and POAG (γ_3_ = 0.5). The frequency of visits was set semi-annually with a 2-year lead-in period. All the other parameters remained constant across all scenarios, with β_0_ = 25, β_1_ = -0.2, β_2_ = 1.0, α_0_ = 0.5, γ_1_ = 0.2, and γ_2_ = 1.0.

**Fig. 2:**
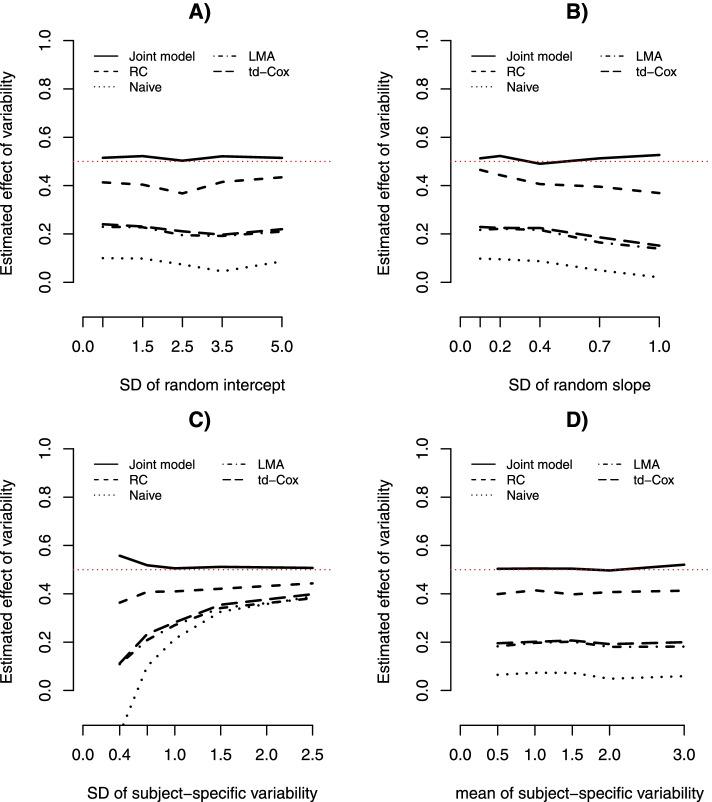
Average estimated effect of biomarker variability on survival outcome ($\widehat{{\gamma }_{3}}$) from the joint model and two-stage methods based on the second simulation, where the dotted red-line represents the true value (γ_3_ = 0.5). **A** $\widehat{{\gamma }_{3}}$ as function of varying SD of random intercept; **B** $\widehat{{\gamma }_{3}}$ as function of varying SD of random slope; **C** $\widehat{{\gamma }_{3}}$ as function of varying SD of within-subject variability; **D** $\widehat{{\gamma }_{3}}$ as function of varying mean of within-subject variability

Figure 2A-D showed the average estimated effect of biomarker variability ($\widehat{{\gamma }_{3}}$) under the above 4 scenarios, respectively. The average estimates of other parameters were also presented in Supplementary Tables 5, 6, 7 and 8, respectively.

- In Scenario 1 (Fig. 2A), Naive showed the largest bias and RC performed the best among the two-stage methods. LMA and td-Cox, having almost identical performance, fell in-between. The results also showed that, at least within the range of specified values, ${\sigma }_{I}$ had little impact on estimating γ_3._ Similar observations were made in the Scenario 4 (Fig. 2D) for the varying ${\mu }_{V}$.
- In Scenario 2 (Fig. 2B), the relative performance of two-stage methods remained the same as above, but the performance of all the methods slightly decreased as ${\sigma }_{S}$ increased.
- In Scenario 3 (Fig. 2C), the accuracy of estimation for all two-stage methods improved as ${\sigma }_{V}$ increased and Naïve converged to LMA and td-Cox in the presence of a strong within-subject variability. When there existed very weak within-subject variability, all methods had a poor performance to detect the effect of variability on outcome—the joint model produced slightly overestimation and all two-stage methods resulted in severe underestimation.

## Discussion

In this paper, using simulated data we compared the relative performance of several two-stage methods in estimating the prognostic effect of biomarker variability on survival outcome. These two-stage methods were chosen for evaluation because they have been widely used in medical applications or thoroughly evaluated in other studies against joint models [2, 3]. Among these methods, the simple approach (Naïve) provides an easy interpretation, requires the least efforts in data manipulation and model fit, and thus gains most popularity in practical use. However, our simulations revealed that it had the worst performance among all the compared methods. Under the scenarios with relatively short longitudinal measurements, for example, the naïve approach resulted in negative association when there is actually no association and suffered from severe underestimation when there exists a moderate association. One reason is that the naïve model fails to control the number of data points in a longitudinal predictor when calculating the subject-specific estimates. Since those subjects with higher risk are more likely to develop event earlier, the number of data points is also associated with clinical outcome. LMA and td-Cox showed improved performance. They provided reliable estimation when there exists no association, but both still suffered from substantial downward bias in the presence of moderate association. Consistent to the previous researches [1–3], the model-based two-stage method (i.e., RC) produced the least bias among all the two-stage methods. In addition, the results showed that, at least within the range of evaluated scenarios, a relatively long series of measurements (i.e., minimum 7 readings) are needed in order to produce an estimation with < 10% bias for the effect of biomarker variability.

In the analysis of longitudinal data (and multi-level data in general), it has been a long tradition to focus on mean level and/or trajectory, while treating within-subject variability (or level-1 variance) as nuisance parameters. The presence of excessive heterogeneity for within-subject variability often serves as a sign of model misspecification, providing evidence of missing important predictor(s) for the mean function of the model [23]. In recent years, however, there is an increasing interest in modeling heterogeneity of level-1 variance. Rast et al. [24] and Goldstein et al. [21] among other relaxed the assumption of homogenous variance in multi-level mixed models, not only modelling the level-1 variance as a log-linear function of subjects’ characteristics but also allowing the association among random effects in the mean and level-1 variance. Leckie et al. [16] summarized the recent development in this field and also reviewed the software options for fitting such extensions. Martins [11] proposed a joint model with flexible links using standard deviation of residuals as a predictor to assess the stability of CD4 counts on survival outcome in patients with HIV/AIDS. Such a joint model allows simultaneously accounting for the measurement errors in both longitudinal and survival processes, uses information more efficiently and leads to unbiased estimates regarding the “true” relationship between longitudinal and survival data.

Despite the recent progress in joint models [10, 11], two-stage methods remain the most popular approach in assessing the prognostic effect of within-subject biomarker variability on clinical outcomes [5–8]. Several factors contribute to the popularity of two-stage approaches. First, these methods can produce tangible descriptive statistics for each individual regarding biomarker variability/trajectory and thus facilitate an easy interpretation. Second, the wealth of theory and software that have been developed for the conventional regression analyses have greatly smoothed the process of model fitting and diagnosis. In addition, graphical techniques such as Kaplan–Meier curves are readily available under a two-stage analysis framework. However, all the two-stage methods share the potential problem that the uncertainty in the estimates from the first stage is not taken into account when assessing their association with clinical outcome, and it is well known that failure to account for the error in covariates can bias parameter estimates downward to null values [25]. Our simulations suggested that these sample-based two-stage methods (i.e., Naïve, LMA, and td-Cox) be used with caution to assess the effect of within-subject variability on future outcomes, unless a relatively long series of longitudinal biomarkers is available for each subject and/or there exists a strong effect size.

Several alternative approaches are available for introducing the association between variability and clinical outcomes. For example, Henderson et al. [26] introduced a within-subject autocorrelation to the longitudinal sub-model to account for “wiggles” over time and they added a frailty into survival sub-model to accommodate any effect that cannot be explained by the shared random intercept and slope. Jiang et al. [27] took a growth mixture model approach and the association between biomarker variability and clinical outcome was introduced via distinct sub-groups (latent classes) as determined by unique longitudinal patterns. In this study, we selected a joint model that directly relates the within-subject variability of longitudinal data as a predictor to the survival outcome [10, 11]. This model possesses some attractive features on modeling the within-subject variability and is relatively straightforward to interpret as comparing to the frailty-model or growth mixture framework. For example, the effect of variability on clinical outcome can be readily quantified as hazard ratio (e.g.,$HR = \exp (\gamma_{3} ){ )}$ and makes it easy to model the effect under the standard framework such as Cox proportional hazards model.

Many potential variants of the aforementioned two-stage methods are also available for assessing the effect of biomarker variability, depending on which summary statistics are used and how the statistics are estimated. For example, one possible method is the “*risk-set RC*” in Sweeting et al. [3]. It is essentially a mixture of LMA and ordinary RC, where a regression calibration is performed at each landmark point. Per reviewer’s comments to distinguish between two-stage methods with and without future information (i.e., using longitudinal measurements only prior to certain time T to estimate the summary statistics at time T), we also explored the potential performance of Naïve and RC without future information via simulated data. As an illustration, the longitudinal data in both methods only included measurements taken during lead-in periods. Therefore, the new methods under consideration were essentially a special case of the LMA and risk-set RC, respectively (i.e., LMA or risk-set RC with a single landmark point at time 0). The results showed that the new Naïve has similar performance as LMA, while the performance of new RC is comparable to that of the ordinary RC (Supplementary Table 3).

In this study, although the simulation studies were performed based on OHTS data, we expect that the observation is generalizable to similar researches in general. One potential limitation of our study is the intensive computational time for joint modelling and RC approach. In the analysis of OHTS data (*N* = 709), for example, the joint model and RC took approximately 40 min when using 3 parallel chains with 50,000 iterations each. In contrast, those sample-based two-stage approaches with standard software took almost no computation time. To make a simulation study feasible, we used a moderate sample size (*N* = 500) with 30,000 iterations, but it still took about 10 min for each replicate. Each simulation therefore only included 100 replicates.

## Conclusion

A relatively long series of longitudinal measurements is required when the effect of biomarker variability is of interest. Model-based two-stage method (regression calibration) and joint modelling are the preferred methods to fulfil this goal. In contrast, sample-based two-stage methods should be used with caution unless there exists a long series of longitudinal measurements and/or strong effect size.

## Supplementary Information

**Additional file 1.**

## Data Availability

The datasets used/analyzed and SAS codes during the current study are available from the corresponding author on reasonable request and with permission of Ocular Hypertension Treatment Study (OHTS).

## Abbreviations

CCT: Central corneal thickness
IOP: Intraocular pressure
LMA: Landmark analysis
OHTS: Ocular Hypertension Treatment Study
POAG: Primary open angle glaucoma
PSD: Pattern standard deviation
RC: Regression calibration
td-Cox: Cox model with time-dependent covariates
VCDR: Vertical cup/disc ratio
