# Supplementary material for: Comparing statistical methods in assessing the prognostic effect of biomarker variability on time-to-event clinical outcomes
Source: BMC Med Res Methodol. 2022 Jul 22;22:201. doi: 10.1186/s12874-022-01686-7 (PMC9308219; doi:10.1186/s12874-022-01686-7)
Supplement: Supplementary file 1 — Additional file 1. [file 12874_2022_1686_MOESM1_ESM.pdf]

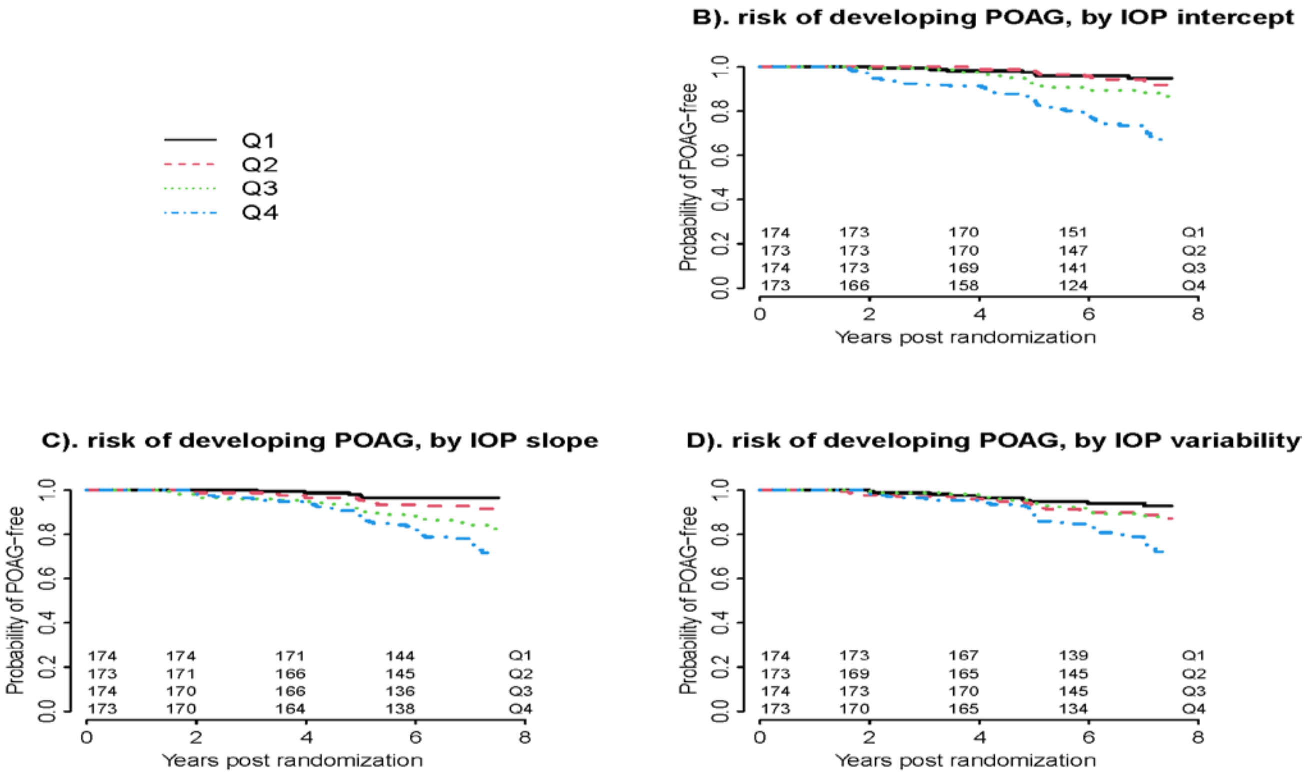

Supplementary Figure 1: An exploratory analysis to assess the association between IOP-derived subject-specific characteristics (i.e., intercept, slope, and variance of residuals from the mixed model incorporating subject-specific variance) and the risk of developing POAG using the OHTS data. **B:** risk of developing POAG by the quartiles of IOP intercept; **C**: risk of developing POAG by the quartiles of IOP slope; **D**: risk of POAG by the quartiles of within-subject IOP variability.

Supplementary Table 1: Average estimated parameters for the sensitivity analysis comparing joint modeling with exponential survival sub-model versus joint modeling with piecewise exponential survival sub-model based on the OHTS data.

Parameters	Piecewise Exponential Joint Model		Exponential Joint Model	
	$\hat{\theta}$	SE	$\hat{\theta}$	SE
**Longitudinal model:**				
**Fixed effects**				
Intercept (β_0)	24.55*	0.108	24.55*	0.109
Slope (β_1)	-0.169*	0.021	-0.171*	0.021
Age (decades) (β_2)	0.244*	0.106	0.235*	0.110
CCT (β_3)	0.045	0.108	0.046	0.111
VCD (β_4)	-0.063	0.110	-0.075	0.105
**Random effects**				
SD of Intercept (σ_I)	2.514*	0.088	2.514*	0.088
SD of slope (σ_S)	0.412*	0.021	0.412*	0.021
SD of variation (σ_V)	0.668*	0.027	0.667*	0.027
Mean of variation (μ_V)	1.641*	0.031	1.640*	0.031
Correlation (ρ_{12})	0.131*	0.060	0.125*	0.060
Correlation (ρ_{13})	0.183*	0.051	0.181*	0.051
Correlation (ρ_{23})	0.235*	0.059	0.235*	0.059
**Survival model:**				
**Baseline factor**				
α_0	--	--	-4.677*	0.175
Age (decades) (α_1)	0.216	0.125	0.223	0.125
CCT (α_2)	-0.639*	0.129	-0.574*	0.128
VCD(α_3)	0.543*	0.142	0.463*	0.135
**Effects of follow-up IOP**				
Intercept (γ_1)	0.226*	0.056	0.228*	0.054
Slope (γ_2)	1.190*	0.514	1.053*	0.405
Variation (γ_3)	0.121	0.228	0.110	0.206

* $P<0.05$;

Supplementary Table 2: Average estimated parameters for the sensitivity analysis comparing Bayesian two-stage model versus non-Bayesian model using the OHTS data.

Parameters	Bayesian Naive		Non-Bayesian Naive	
	$\hat{\theta}$	SE	$\hat{\theta}$	SE
**Survival model:**				
**Baseline factor**				
Age (decades) (α_1)	0.079	0.106	0.082	0.107
CCT (α_2)	-0.680*	0.113	-0.672*	0.110
VCD(α_3)	0.558*	0.112	0.549*	0.111
**Effects of follow-up IOP**				
Intercept (γ_1)	0.197*	0.038	0.212*	0.034
Slope (γ_2)	0.052	0.058	0.038	0.054
Variation (γ_3)	-0.052	0.095	-0.052	0.094

* P<0.05; Naïve: simple OLS two-stage model

Supplementary Table 3: Average of estimated effect of biomarker variability ($\widehat{\gamma_3}$) on survival outcome and its standard error (SE) from two-stage methods with and without post-baseline longitudinal measurements.

Simulated scenarios					Naïve-new		LMA		RC-new		RC	
Frequency of visits	Min #visits (lead-in time)	β_1	γ_3	Scenario#	$\widehat{\gamma_3}$	SE	$\widehat{\gamma_3}$	SE	$\widehat{\gamma_3}$	SE	$\widehat{\gamma_3}$	SE
Semi-annually	3 (1-yr)	0.0	0.0	1	0.003	0.003	0.002	0.034	0.026	0.186	0.039	0.129
			0.5	2	0.036	0.031	0.067	0.038	0.447	0.185	0.372	0.128
		-0.5	0.0	3	0.001	0.030	0.002	0.035	-0.012	0.189	0.018	0.127
			0.5	4	0.041	0.032	0.074	0.039	0.445	0.177	0.419	0.126
	7 (3-yr)	0.0	0.0	5	-0.012	0.069	-0.019	0.081	0.018	0.134	0.044	0.117
			0.5	6	0.222	0.072	0.243	0.085	0.458	0.130	0.483	0.115
		-0.5	0.0	7	0.006	0.070	-0.014	0.080	-0.003	0.132	0.003	0.117
			0.5	8	0.220	0.072	0.243	0.083	0.478	0.131	0.452	0.121
Quarterly	5 (1-yr)	0.0	0.0	9	-0.006	0.057	-0.006	0.068	0.012	0.148	0.002	0.114
			0.5	10	0.165	0.061	0.218	0.073	0.450	0.144	0.452	0.115
		-0.5	0.0	11	-0.004	0.058	0.004	0.068	-0.006	0.147	0.017	0.112
			0.5	12	0.149	0.061	0.205	0.073	0.437	0.141	0.413	0.113
	13 (3-yr)	0.0	0.0	13	-0.015	0.084	-0.014	0.097	0.012	0.115	0.008	0.107
			0.5	14	0.298	0.085	0.313	0.098	0.488	0.117	0.474	0.109
		-0.5	0.0	15	-0.014	0.084	-0.024	0.096	0.008	0.116	0.001	0.108
			0.5	16	0.301	0.085	0.314	0.098	0.457	0.117	0.462	0.108

Naïve-new: simple OLS with baseline longitudinal measurements only; LMA: landmark analysis;
RC-new: regression calibration with baseline longitudinal measurements only; RC: ordinary regression calibration.

Exploratory analysis of OHTS data: We also conducted an exploratory analysis using the OHTS data to answer a question clinicians may have, "**How many follow-up visits would be needed for an accurate estimate of within-subject IOP variability**?" To answer this question, we examined how much improvement, if any, each additional follow-up visit made in the accuracy of the estimating each predictor. We used on a subset of IOP data from 478 OHTS participants who did not develop POAG during study and had a full set of 13 IOP measurements taken at 6-month intervals. A series of OLS models were fitted to each participant at each visit to estimate the summary statistics (intercept, slope, and SD of residuals) in a similar way as that in the LMA/tdCox models. We calculated the coefficient of variation (CV) for intercept and SD of residuals and plotted its value at each visit (**Supplementary Figure 2**). Smaller CV indicated more precise estimation. As the plot showed, as few as 3 IOP measurements can provide a reliable estimate of intercept IOP (CV=0.13). For the SD of residuals, by contrast, the coefficient of variation was 0.86 with 3 IOP measurements. The CV showed a rapid decrease initially, but had modest improvement after 7 or 8 measurements and failed to reach CV=0.13 even after 13 measurements. Since many clinical trials only have a handful of longitudinal measurements in most subjects, the issue of measurement error in such sample-based statistics can be substantial.

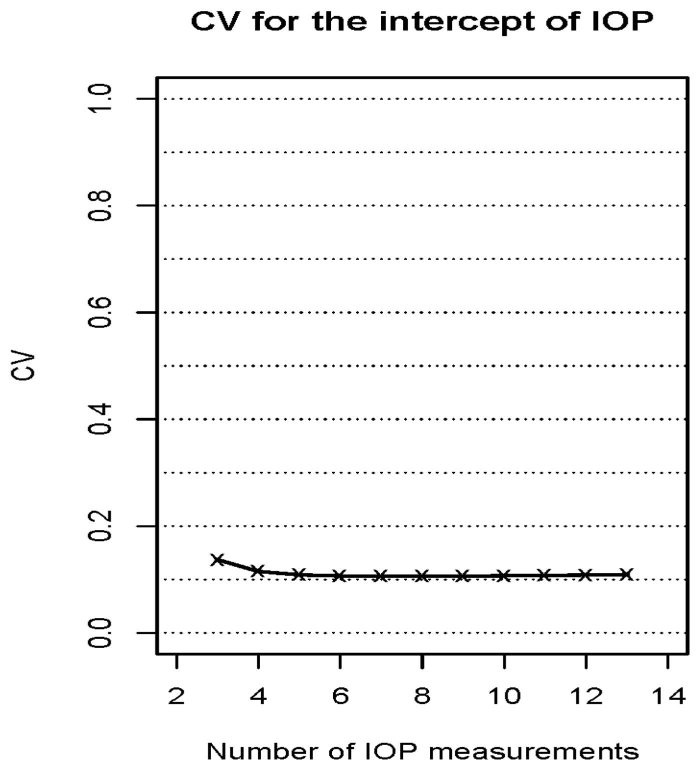

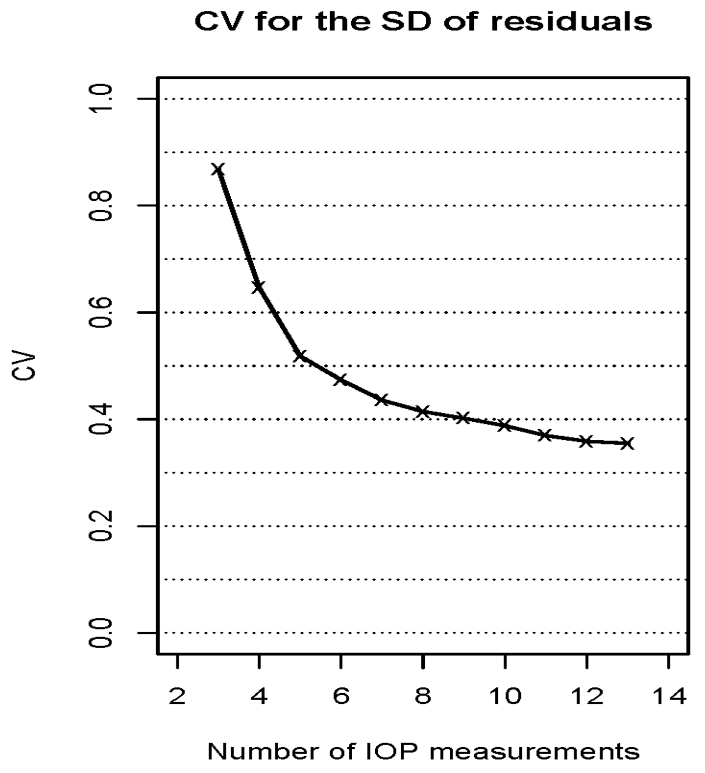

Supplementary Figure 2: Coefficients of variation (CV) for characteristics derived from the longitudinal IOPs in N=478 OHTS participants who had at least 13 IOPs taken during follow-up, where the characteristics (Intercept, SD of residuals) at each visit are based on simple OLS models fitted to each subject using IOP accumulated up to the given visit. **Left:** CV of intercept against number of measurements; **Right:** CV for SD of residuals against number of measurements.

Supplementary Table 4: Average estimated parameters in the survival (sub-) models from joint modelling and two-stage methods based on the first simulation, where 16 scenarios were simulation with different number of longitudinal measurements.

Scenario#	Naïve approach				Landmark analysis				Time-dependent Cox			
	γ_1	γ_2	γ_3	α_1	γ_1	γ_2	γ_3	α_1	γ_1	γ_2	γ_3	α_1
[1]	0.163	0.153	-0.354	0.282	0.137	-0.026	0.002	0.307	0.143	-0.019	0.001	0.318
[2]	0.148	0.113	-0.238	0.285	0.132	-0.029	0.066	0.307	0.137	-0.028	0.076	0.315
[3]	0.154	0.138	-0.355	0.307	0.133	-0.028	0.002	0.336	0.137	-0.024	0.003	0.341
[4]	0.152	0.121	-0.226	0.290	0.132	-0.026	0.074	0.308	0.136	-0.028	0.088	0.321
[5]	0.173	0.424	-0.129	0.311	0.143	0.078	-0.019	0.322	0.148	0.108	-0.018	0.329
[6]	0.164	0.371	0.196	0.304	0.137	0.081	0.243	0.313	0.143	0.100	0.259	0.323
[7]	0.169	0.428	-0.121	0.312	0.137	0.083	-0.014	0.326	0.145	0.113	-0.005	0.330
[8]	0.164	0.352	0.191	0.308	0.137	0.072	0.243	0.315	0.141	0.078	0.258	0.325
[9]	0.179	0.220	-0.214	0.296	0.153	-0.032	-0.006	0.308	0.158	-0.026	-0.012	0.315
[10]	0.171	0.189	0.122	0.295	0.144	-0.028	0.218	0.310	0.150	-0.029	0.232	0.316
[11]	0.176	0.231	-0.222	0.298	0.147	-0.028	-0.004	0.320	0.154	-0.024	-0.013	0.318
[12]	0.175	0.202	0.102	0.278	0.152	-0.031	0.205	0.283	0.156	-0.027	0.222	0.295
[13]	0.179	0.583	-0.080	0.302	0.147	0.190	-0.014	0.311	0.153	0.216	-0.025	0.318
[14]	0.177	0.506	0.299	0.297	0.151	0.156	0.313	0.303	0.155	0.177	0.321	0.311
[15]	0.184	0.587	-0.074	0.309	0.153	0.190	-0.024	0.322	0.159	0.227	-0.023	0.329
[16]	0.174	0.538	0.302	0.299	0.144	0.175	0.314	0.311	0.150	0.196	0.321	0.315

Scenario#	Regression Calibration				Joint Model			
	γ_1	γ_2	γ_3	α_1	γ_1	γ_2	γ_3	α_1
[1]	0.187	0.417	0.039	0.455	0.200	1.004	0.011	0.504
[2]	0.173	0.367	0.372	0.472	0.199	1.034	0.507	0.513
[3]	0.187	0.416	0.018	0.467	0.209	1.094	-0.017	0.532
[4]	0.172	0.385	0.419	0.475	0.201	1.034	0.528	0.523
[5]	0.183	0.675	0.044	0.490	0.199	1.031	0.001	0.501
[6]	0.181	0.656	0.483	0.483	0.207	1.088	0.499	0.506
[7]	0.188	0.631	0.003	0.485	0.196	0.991	-0.010	0.509
[8]	0.185	0.608	0.452	0.477	0.212	1.053	0.521	0.510
[9]	0.190	0.509	0.002	0.482	0.195	0.986	-0.015	0.516
[10]	0.178	0.479	0.452	0.492	0.203	1.013	0.489	0.498
[11]	0.190	0.469	0.017	0.486	0.199	1.045	0.011	0.526
[12]	0.179	0.493	0.413	0.480	0.204	1.020	0.524	0.513
[13]	0.186	0.768	0.008	0.485	0.199	0.966	0.00 5	0.515
[14]	0.180	0.759	0.474	0.472	0.202	1.045	0.515	0.519
[15]	0.191	0.753	0.001	0.486	0.202	1.036	-0.005	0.507
[16]	0.182	0.704	0.462	0.470	0.207	1.003	0.519	0.504

True values: $\gamma_1 = 0.2$; $\gamma_2 = 1.0$; $\alpha_1 = 0.5$; $\gamma_3 = 0$ (under Scenarios 1, 3, 5, 7, 9, 11, 13, and 15) and $\gamma_3 = 0.5$ (otherwise);

γ_1, γ_2, γ_3: effects of random intercept, slope and within-subject variability, respectively

Supplementary Table 5: Average estimated parameters for survival (sub-) models from the joint modelling and two-stage methods based on the second simulation, where 5 different scenarios were simulated with varying standard deviation (SD) of random intercept.

Scenario#	Naïve approach				Landmark analysis				Time-dependent Cox			
	γ_1	γ_2	γ_3	α_1	γ_1	γ_2	γ_3	α_1	γ_1	γ_2	γ_3	α_1
[1]	0.054	0.327	0.100	0.422	-0.042	0.140	0.230	0.508	-0.035	0.136	0.240	0.510
[2]	0.147	0.305	0.098	0.326	0.097	0.018	0.228	0.360	0.100	0.033	0.231	0.369
[3]	0.165	0.289	0.074	0.314	0.138	0.001	0.195	0.328	0.142	0.004	0.211	0.335
[4]	0.172	0.247	0.045	0.299	0.157	-0.024	0.192	0.308	0.160	-0.017	0.197	0.310
[5]	0.181	0.234	0.086	0.279	0.170	-0.027	0.210	0.279	0.175	-0.023	0.220	0.286

Scenario#	Regression Calibration				Joint Model			
	γ_1	γ_2	γ_3	α_1	γ_1	γ_2	γ_3	α_1
[1]	0.137	0.525	0.413	0.493	0.164	1.048	0.515	0.512
[2]	0.181	0.447	0.404	0.472	0.203	1.003	0.522	0.509
[3]	0.189	0.329	0.367	0.462	0.204	0.998	0.503	0.513
[4]	0.186	0.357	0.415	0.492	0.203	1.078	0.521	0.513
[5]	0.190	0.403	0.434	0.468	0.210	0.934	0.514	0.527

True values: $\gamma_1 = 0.2$; $\gamma_2 = 1.0$; $\gamma_3 = 0.5$; $\alpha_1 = 0.5$; γ_1, γ_2, γ_3: effects of random intercept, slope and within-subject variability, respectively

Supplementary Table 6: Average estimated parameters for survival (sub-) models from the joint modelling and two-stage methods based on the second simulation, where 5 different scenarios were simulated with varying standard deviation (SD) of random slope.

Scenario#	Naïve approach				Landmark analysis				Time-dependent Cox			
	γ_1	γ_2	γ_3	α_1	γ_1	γ_2	γ_3	α_1	γ_1	γ_2	γ_3	α_1
[1]	0.177	0.008	0.098	0.302	0.169	-0.133	0.217	0.315	0.168	-0.122	0.229	0.315
[2]	0.172	0.098	0.095	0.316	0.157	-0.099	0.222	0.327	0.161	-0.092	0.225	0.332
[3]	0.168	0.320	0.087	0.307	0.141	0.002	0.217	0.317	0.144	0.018	0.225	0.329
[4]	0.153	0.515	0.050	0.312	0.101	0.173	0.165	0.329	0.108	0.194	0.186	0.349
[5]	0.145	0.580	0.020	0.296	0.083	0.289	0.139	0.332	0.086	0.309	0.152	0.343

Scenario#	Regression Calibration				Joint Model			
	γ_1	γ_2	γ_3	α_1	γ_1	γ_2	γ_3	α_1
[1]	0.192	-0.409	0.465	0.502	0.216	0.506	0.512	0.532
[2]	0.182	-0.046	0.443	0.484	0.213	0.930	0.522	0.513
[3]	0.181	0.322	0.406	0.480	0.210	1.005	0.490	0.519
[4]	0.170	0.516	0.395	0.467	0.200	0.988	0.513	0.497
[5]	0.152	0.517	0.369	0.428	0.199	1.002	0.526	0.511

True values: $\gamma_1 = 0.2$; $\gamma_2 = 1.0$; $\gamma_3 = 0.5$; $\alpha_1 = 0.5$; γ_1, γ_2, γ_3: effects of random intercept, slope and within-subject variability, respectively

Supplementary Table 7: Average estimated parameters for survival (sub-) models from the joint modelling and two-stage methods based on the second simulation, where 5 different scenarios were simulated with varying standard deviation (SD) of within-subject variability.

Scenario#	Naïve approach				Landmark analysis				Time-dependent Cox			
	γ_1	γ_2	γ_3	α_1	γ_1	γ_2	γ_3	α_1	γ_1	γ_2	γ_3	α_1
[1]	0.182	0.372	-0.178	0.307	0.156	0.002	0.109	0.322	0.161	0.033	0.112	0.328
[2]	0.170	0.283	0.101	0.302	0.143	-0.008	0.209	0.309	0.147	-0.004	0.234	0.324
[3]	0.157	0.216	0.213	0.311	0.130	-0.021	0.270	0.330	0.132	-0.016	0.282	0.336
[4]	0.122	0.032	0.326	0.331	0.103	-0.055	0.341	0.342	0.103	-0.057	0.355	0.348
[5]	0.034	-0.013	0.389	0.407	0.038	-0.030	0.382	0.389	0.029	-0.022	0.399	0.410

Scenario#	Regression Calibration				Joint Model			
	γ_1	γ_2	γ_3	α_1	γ_1	γ_2	γ_3	α_1
[1]	0.183	0.392	0.363	0.481	0.206	1.080	0.558	0.516
[2]	0.192	0.260	0.408	0.461	0.206	1.071	0.518	0.514
[3]	0.176	0.287	0.408	0.472	0.204	1.005	0.505	0.504
[4]	0.175	0.393	0.421	0.473	0.201	1.100	0.511	0.512
[5]	0.174	0.436	0.443	0.458	0.207	0.939	0.507	0.507

True values: $\gamma_1 = 0.2$; $\gamma_2 = 1.0$; $\gamma_3 = 0.5$; $\alpha_1 = 0.5$; γ_1, γ_2, γ_3: effects of random intercept, slope and within-subject variability, respectively

Supplementary Table 8: Average estimated parameters for survival (sub-) models from the joint modelling and two-stage methods based on the second simulation, where 5 different scenarios were simulated with varying mean of within-subject variability.

Scenario#	Naïve approach				Landmark analysis				Time-dependent Cox			
	γ_1	γ_2	γ_3	α_1	γ_1	γ_2	γ_3	α_1	γ_1	γ_2	γ_3	α_1
**[1]**	0.175	0.420	0.065	0.285	0.155	0.118	0.183	0.291	0.159	0.120	0.196	0.299
**[2]**	0.165	0.278	0.073	0.294	0.144	0.034	0.197	0.304	0.148	0.030	0.202	0.310
**[3]**	0.156	0.183	0.073	0.294	0.134	-0.018	0.202	0.302	0.138	-0.019	0.207	0.311
**[4]**	0.141	0.118	0.049	0.305	0.117	-0.042	0.181	0.311	0.120	-0.039	0.192	0.325
[5]	0.106	0.021	0.059	0.332	0.085	-0.052	0.182	0.336	0.087	-0.054	0.200	0.355

Scenario#	Regression Calibration				Joint Model			
	γ_1	γ_2	γ_3	α_1	γ_1	γ_2	γ_3	α_1
[1]	0.186	0.594	0.398	0.472	0.205	1.042	0.504	0.519
[2]	0.185	0.507	0.414	0.452	0.198	1.004	0.505	0.506
[3]	0.179	0.376	0.397	0.462	0.203	1.001	0.504	0.518
[4]	0.165	0.342	0.407	0.464	0.206	0.945	0.497	0.515
[5]	0.161	-0.047	0.412	0.459	0.211	0.731	0.520	0.515

True values: $\gamma_1 = 0.2$; $\gamma_2 = 1.0$; $\gamma_3 = 0.5$; $\alpha_1 = 0.5$; γ_1, γ_2, γ_3: effects of random intercept, slope and within-subject variability, respectively

Codes for regression calibration (RC) and joint model:

```
###################################
# 1. Codes of RC model for the OHTS data
###################################

mymodel <- function(){

  for (i in 1:N) {
     ### For longitudinal model with IOP change over time;
     ### 5 fixed effects:  Intercept, Slope, Age, CCT, VCD;
      for (j in 1:M) {
        mu.yy[i,j] <-  RE.i[i,1] + RE.i[i,2]*tt[j]   +
                beta.y[1]*age[i] + beta.y[2]*cct[i] + beta.y[3]*vcd[i]
        yy[i,j] ~ dnorm(mu.yy[i,j], tau.vi[i])
     }
     tau.vi[i] <- 1/exp(RE.i[i,3])
     RE.i[i, 1:3] ~ dmnorm(RE.mu[], RE.Sigma[,])
  }

  ### variance-covariance matrix for random effects {I_i, S_i, U_i}
  RE.var[1:3,1:3] <- inverse(RE.Sigma[,])

  ### mean levels for patient-specific intercept, slope, and variability
  beta0<- RE.mu[1]
  beta1<- RE.mu[2]
  var.alpha<- RE.mu[3]

  ### priors
  beta.y[1:n.para.y] ~ dmnorm( beta.y.mu[], Sigma.y[,])
  RE.mu[1:3] ~ dmnorm(rand.mean[], rand.prec[,])
```

```
   RE.Sigma[1:3, 1:3] ~ dwish(Omega[ , ], 3)
}

##############################################
# 2. Codes of Joint Model for the OHTS data
##############################################

mymodel <- function(){

   for (i in 1:N) {
       ### For longitudinal sub-model of IOP change;
       ### 5 fixed effects: Intercept, Slope, Age, CCT, VCD;
       for (j in 1:M) {
          mu.yy[i,j] <-  RE.i[i,1] + RE.i[i,2]*tt[j]  +
                  beta.y[1]*age[i] + beta.y[2]*cct[i]   +  beta.y[3]*vcd[i]
          yy[i,j] ~ dnorm(mu.yy[i,j], tau.vi[i])
       }
       tau.vi[i] <- 1/exp(RE.i[i,3])

     ### For survival sub-model with piece-wise exponential model
     ### 6 fixed effects: Age, CCT, VCD, IOP mean, IOP slope, IOP SD;
     for (k in 1:K) {
        d[i, k]<-(fail[i])*step(tee[i]-ht[k])*step(ht[k+1]-tee[i])
        delta[i, k]<- (min(tee[i], ht[k+1]) - ht[k])*step(tee[i]-ht[k])
        d[i,k] ~ dpois(mu[i, k])
        mu[i, k] <- delta[i,k]* haz0[k] * exp(
                     r1*(RE.i[i,1] - RE.mu[1])     +     r2*(RE.i[i,2] - RE.mu[2])     +
                     r3*(RE.i[i,3] - RE.mu[3])     +     r4*age[i] + r5*cct[i]  + r6*vcd[i] )
        }
      RE.i[i, 1:3] ~ dmnorm(RE.mu[], RE.Sigma[,])
   }
```

```
    ### K parameters for baseline hazard function
    for (k in 1:K) {
     haz0[k] ~ dgamma (0.1, 10)
    }

    ### variance-covariance matrix for random effects
    RE.var[1:3,1:3] <- inverse(RE.Sigma[,])
    sd.int.out<-sqrt(RE.var[1,1])
    sd.slope.out <- sqrt(RE.var[2,2])
    sd.logvi.out <- sqrt(RE.var[3,3])
    rho12.out <-    RE.var[1,2]/(sqrt(RE.var[1,1])*sqrt(RE.var[2,2]))
    rho13.out <-    RE.var[1,3]/(sqrt(RE.var[1,1])*sqrt(RE.var[3,3]))
    rho23.out <-    RE.var[2,3]/(sqrt(RE.var[3,3])*sqrt(RE.var[2,2]))

    ### mean levels for patient-specific intercept, slope, and variability
    beta0<- RE.mu[1]
    beta1<- RE.mu[2]
    var.alpha<- RE.mu[3]

    ### priors
    beta.y[1:n.para.y] ~ dmnorm( beta.y.mu[], Sigma.y[,])
        r1 ~ dnorm(0, 0.01)
        r2 ~ dnorm(0, 0.01)
        r3 ~ dnorm(0, 0.01)
        r4 ~ dnorm(0, 0.01)
        r5 ~ dnorm(0, 0.01)
        r6 ~ dnorm(0, 0.01)
    RE.mu[1:3] ~ dmnorm(rand.mean[], rand.prec[,])
    RE.Sigma[1:3, 1:3] ~ dwish(Omega[ , ], 3)
}
```
